# Description of helminthic fauna of *Trachemys scripta* (Thunberg in Schoepf, 1792) in a protected area in Northern Italy: evidence of co-introduction of non-native helminth species

**DOI:** 10.3389/fvets.2026.1798584

**Published:** 2026-04-24

**Authors:** Erica Marchiori, Paola Beraldo, Cinzia Tessarin, Elisabetta Ferraro, Stefano Pesaro, Federica Marcer

**Affiliations:** 1 Department of Animal Medicine, Production and Health, University of Padova, Legnaro (PD), Italy; 2 Department of Agricultural, Food, Environmental and Animal Sciences, University of Udine, Udine (UD), Italy

**Keywords:** co-introduction, emydidae, freshwater turtle, biological invasion, parasites

## Abstract

Biological invasions are occurring at an unprecedented rate around the globe. Their impact on biodiversity is mediated both by direct competition with native species and by the alteration of transmissible disease dynamics. The red eared slider *Trachemys scripta* is considered one of the most successful invasive species across Europe, and has proved to be a stronger competitor with the native freshwater turtle *Emys orbicularis*. Nevertheless, few studies have explored parasites of *T. scripta* in the countries of introduction. Aiming to explore the taxonomic helminth composition of *T. scripta* and assess potential spillover risks to *E. orbicularis*, a population of red eared sliders was sampled from a protected area in Northeastern Italy, where the two turtle species co-exist. Sixty-two animals were humanely euthanised and submitted to complete parasitological investigation. Morphometric and molecular data were combined to identify the helminths to species level. Overall, 68.4% of the animals were positive for helminths. Three species were recovered from the gastrointestinal tract, namely *Telorchis corti* (Digenea: Telorchiidae), *Polystomoides oris* (Monogenea: Polystomatidae), *Serpinema microcephalus* (Nematoda: Camallanidae) and one from the urinary bladder, *Polystomoides orbicularis*. Three of the four species retrieved are considered exotic, two of them already reported in Southern Europe also in native species. Pancreatic lesions were observed in association to *S. microcephalus*, demonstrating enhanced pathology in this host. Studies on *E. orbicularis* are recommended to shed light on patterns of parasite exchange among the two emydid species. Additionally, a wider survey across the national territory is advised to further explore the helminth composition hosted by this invasive species.

## 1 Background

The continuous proliferation and dispersal of non-native species create unique challenges in evaluating the present state of biological invasions. The number of both vegetal and animal species expanding beyond their native range is continuously increasing, and providing accurate updates is a challenging task (1). From the voluntary, transcontinental pet trade to the involuntary transport of aquatic organisms via ballast water, the spread of animal species worldwide beyond their original territories represents a major threat to biodiversity (2–4). Marine and freshwater habitats have been deeply transformed by invasive alien species belonging to a number of different taxa, which, depending on their trophic role, are able to disrupt the food web from both its base and apex (5, 6). Moreover, co-transport of pathogens in the wake of their natural hosts represents a silent threat (6–8). The impact of newly introduced species on native fauna is indeed to be measured not only in terms of direct competition but also of the alteration of transmissible disease dynamics. Spillover of co-introduced parasites into autochthonous species can result into highly virulent infections due to the absence of co-evolution. On the other hand, invasive species may act as vectors or reservoirs of local parasites, amplifying the abundance of infective forms (spillback) (8–11). Finally, the invasive species may lose its parasite, in terms of prevalence, diversity and burden, as a result of introduction, facilitating its growth and spread (enemy release) (9, 12). Direct and indirect competition may become even more relevant when they relate to endangered local species already threatened by environmental changes and population fragmentation.

Among freshwater reptiles, the population decline of the European pond slider *Emys orbicularis* (Chelonia: Emydidae) has been mainly attributed to habitat loss and landscape disruption. Concomitantly, this decline is further exacerbated by the co-existence of the exotic invasive species *Trachemys scripta* (13, 14) in the same fragile habitat. The presence of this species, native to Northern and Central America, became increasingly common in Europe and Italy after its widespread marketing as a pet animal since the ‘70s. Following an illegal release into the wild, the species has spread into freshwater environments, becoming a stronger competitor with the autochthonous European pond slider (15, 16). Moreover, parasite spillover events from *T. scripta* to the European pond turtle *E. orbicularis* have been reported in the last few years in Southern Europe (17–22), some of which showing relevant effect on the new, naïve host. Exotic monogenean species of the family Polystomatidae, living in conjunctival sac, urinary bladder or oral cavity of chelonians, have been found in the autochthonous species *E. orbicularis* and *Mauremys leprosa* in Spain and France, demonstrating a previously unexpected ability of host-switching within the family Cryptodira (18–20, 22). Most remarkably, outbreaks of spirorchidiasis, caused by a Nearctic species of cardiovascular flukes, *Spirorchis* spp. have recently been reported from Northern Spain and Switzerland (17, 21). Severe parasitic disease and fatal outcome were reported in *E. orbicularis* in both cases, supporting the hypothesis that increased virulence is typical of infections caused by non-co-evolved parasites in novel hosts. In Italy, descriptions of the parasitofauna of both autochthonous and invasive turtle species are lacking, despite ongoing conservation projects aimed at preventing further decline of *E. orbicularis* population.

The present study describes for the first time in Italy the helminth composition of *T. scripta.* The area under consideration is a protected natural site wherethe exotic species co-occurs with *E. orbicularis*, thereby increasing the risk of interspecies parasite transmission. Potential alterations in parasitic disease dynamics among emydid species inhabiting Italian marshes are explored, as is the potential for diversity loss.

## 2 Materials and methods

### 2.1 Parasite sampling

Samples were obtained during an eradication campaign of the allochthonous turtle species *Trachemys scripta* in Friuli Venezia Giulia region, Northeastern Italy, during spring 2022 and 2023 and summer 2024. In accordance with Italian legislation (D. lgs. 230/2017, art.22), eradication of the species is to be achieved in natural protected areas where it occurs sympatrically with the European pond turtle, *E. orbicularis*. The protected site largely coincides with the SAC/SPA “Foce dell’Isonzo – Isola della Cona” IT3330005, a Natura 2000 Network site established pursuant to European Directives 43/92 “Habitats” and 147/09 “Birds,” and is included in the Wetlands of International Importance under the Ramsar Convention (16A02517).

The turtles were captured using basking traps, left on site for 1 week and checked daily (23) and humanely euthanized using sedation followed by thiopental sodium injection (24). In the eventuality of accidental trapping of non-target species, these were immediately released and returned in the environment.

Necropsies were performed at the Department of Agri-Food, Environmental and Animal Sciences, University of Udine, as described by Shilton et al. (25). Before dissection, turtles were identified to species and subspecies level based on morphological characteristics. For each specimen, curved carapace length was taken using a tape measure to estimate maturity (adult, male >10 cm, female >16 cm), along with body mass (g) and sex determination (26). Subsequently, the plastron was removed to access the celomatic cavity. Lungs, kidneys, liver and spleen were dissected and observed at the stereomicroscope for the presence of spirorchiid eggs or related lesions; heart and major vessels were opened longitudinally, carefully observed and washed in tap water in Petri dishes for the research of spirorchiid flukes. The entire gastrointestinal tract was tightened at the extremities during extraction from the visceral cavity, then opened and washed in tap water while scraping the walls, to collect any parasite that may have adhered to the mucosal layer. Feces were collected separately and copromicroscopic examination was performed following Marchiori et al. (27). Once the urinary bladder was removed, its content and walls were examined at the stereomicroscope for the presence of monogenean polystomes. After sedimentation in conical flasks, the content of gastrointestinal tract was observed under the stereomicroscope. Helminths were counted, collected and stored in ethanol 70% for further morphological and molecular analyses. Specimens were then clarified in glycerol 50%, and identification was attained by observation of morphometric features at the optic microscope at 4-40X using keys in literature (20, 28–32). Prevalence and mean intensity were calculated following Bush et al. (33) for the different helminth taxa using the software Quantitative Parasitology. The 95% confidence intervals for prevalence and mean intensity were calculated using the Clopper-Pearson method and the bias-corrected accelerated bootstrap method with 2000 replications, respectively (34).

During necropsy, tissue samples were collected from organs exhibiting grossly visible lesions, fixed in 4% buffered formaldehyde, and automatically processed for histopathological evaluation.

### 2.2 Molecular identification

Helminths were removed from 70% ethanol, washed in phosphate-buffered saline, and left to dry overnight; DNA was then extracted using NucleoSpin® Tissue Kit (Macherey-Nagel, Germany), according to the manufacturer’s instructions. A PCR amplifying the 28S rDNA fragment was performed on all samples using the primers LSU5 and 1500R (35) (Table 1). Furthermore, a portion of the mtDNA cytochrome oxidase1 (COX1) was amplified using the universal primers CO1490 and HCO2198 (36) for nematodes, primers L-CO1p and H-COX1p2 (37) for monogeneans, and primers JB3 and JB4.5 (38) for trematodes.

**Table 1:** List of the primers used for amplification and sequencing rDNA 28S and mtCOI gene.

Target	Primers	Sequence	bp	Source
28S	LSU-5	5′-TAG GTC GAC CCG CTG AAY TTA AGC A-3′	1,300	Olson et al. (35)
	1500R	5’-GCT ATC CTG AGG GAA ACT TCG-3′		
COX1	CO1490	5’-GGTCAACAAATCATAAAGATATTGG-3’	710	Folmer et al. (36)
	HCO2198	5’-TAAACTTCAGGGTGACCAAAAAATCA-3’		
COX1	L-CO1p	5′ – TTTTTTGGGCATCCTGAGGTTTAT −3’	440	Littlewood et al. (37)
	H-COX1p2	5’-TAAAGAAAGAACATAATGAAAATG-3′		
COX1	JB3	5’-TTTTTTGGGCATCCTGAGGTTTAT-3’	450	Pauly et al. (38)
	JB4.5	5’-TAAAGAAAGAACAT–AATGAAAATG-3’		

PCR products were visualized in 2% agarose gels and the amplicons were purified with ExoSAP (Thermo Fisher Scientific Inc., USA) and sequenced in both directions (Macrogen Europe, The Netherlands). DNA sequence chromatograms were evaluated using the software ChromasPro version 2.4.3 (Technelysium Pty Ltd., Austria). *Consensus* sequences were assembled with the SeqMan program available in the DNAstar package and compared with the non-redundant database in GenBank using BLASTn (39).

## 3 Results

Overall, 62 animals were collected and submitted to complete parasitological examination. The sample comprised both mature (*n =* 50, 14 males and 36 females) and immature animals (*n =* 12, 8 females, 4 males). Overall, 69.4% (43/62) of the animals were positive for at least one helminth species, with mean species richness per turtle being 0.87.

Three helminth species belonging to the Phylum Nematoda and Platyhelminthes (Classes Trematoda [subclass Digenea] and Monogenea) were collected and identified from the gastrointestinal tract. Furthermore, one Monogenea species was identified from the urinary bladder. Table 2 reports epidemiological indeces for the different taxa.

**Table 2:** Infection parameters of helminth species retrieved from *Trachemys scripta* (*n =* 62).

Species	Site of infection	N° positive (P %) [95%CI]	Mean intensity [95%CI]	Intensity range
Digenea				
*Telorchis corti*	Intestine	28/62 (45.2) [32.5–58.3]	33.7 [15.4–82.5]	1–371
Monogenea				
*Polystomoides oris*	Stomach	4/62 (6.4) [1.8–15.7]	3.3 [1.5–6]	1–7
*Polystomoides orbicularis*	Urinary bladder	9/62 (14.5) [6.9–25.8]	5.1 [1.22–15.6]	1–31
Nematoda				
*Serpinema microcephalus*	Intestine	13/62 (21.0) [11.7–33.2]	5.5 [3.0–9.7]	1–20

P, prevalence; 95%CI, 95% confidence interval.

### 3.1 Morphological identification of helminths

#### 3.1.1 Trematoda: Digenea

**Figure 1:**
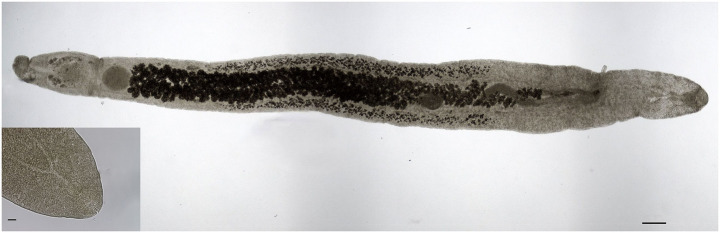
*Telorchis corti* isolated from intestines of *Trachemys scripta*. Bar = 250 μm. Inset: Detail of the anterior end showing spiny cuticle, oral sucker, pharynx, and the short esophagus. Bar = 50 μm.

Overall, 994 trematodes of the genus *Telorchis* Lühe, 1899 were found in the intestinal content of 28 animals. Identification to genus level was achieved by observing conserved characters, i.e., position of the testis at the end of the body, ovary pretesticular and separated from testes by the coiled uterus (40). The following morphometric features were observed and used for identification of our isolate as species *Telorchis corti* Stunkard, 1915 (Figure 1 and Table 3): filiform body shape, with length to width ratio 11:1; body elongated; oral sucker lappets absent, presence of a short esophagus separating pharynx and caecal bifurcation; long and thin cirrus sac, terminating just anteriorly to ovary and without bulb at the distal end of the metraterm. The ovary is closer to the acetabulum than the testes, and spherical in shape. Vitelline follicles occur in two continuous longitudinal rows on either side, starting anteriorly to the ovary. Testis are spherical or subspherical in tandem, and caeca terminate posteriorly to the distal testis.

**Table 3:** Morphometric parameters (μm) of 13 specimens of *Telorchis corti*, collected from *Trachemys scripta* (*n =* 10).

Characteristics	Min-max (*n*) (mean; DS)
Body Length	2,318–7,315 (10) (5,022; 1,476)
Body Width	274–753 (9) (532; 134)
Oral Sucker length	87–209 (8) (132; 34)
Oral Sucker Width	112–152 (9) (128; 12)
Pharynx length	41–96 (6) (70; 17)
Pharynx width	64–120 (8) (81; 17)
Esophagus length	94–198 (3) (134; 46)
Ventral Sucker length	92–126 (7) (117; 21)
Ventral Sucker width	82–169 (7) (122; 30)
Anterior Testis Length	219–307 (7) (260; 31)
Anterior Testis Width	174–270 (7) (242; 35)
Posterior Testis Length	218–324 (7) (267;34)
Posterior Testis Width	158–304 (7) (245; 44)
Ovary Length	123–245 (9) (173; 41)
Ovary Width	119–218 (9) (162; 39)
Cirrus Sac Length	1,203–1,295 (2) (1,249;46)
Eggs	29-36×18-19 (9) (34×19; 2.22×1.23)

n, sample size.

*Remarks*: our isolate differs from other species of the genus from Europe by having a long cirrus, coming to ¾ of the distance to the ovary but not reaching it, as in *Telorchis solivagus* Odhner, 1902 and in *Telorchis aculeatus* (von Linstow, 1879) (41). In the latter species, Stunkard (1916) also noted that vitellaria do not expand as much anteriorly and posteriorly as in *T corti* and eggs are bigger (46 × 19 versus 26–30 × 12–16 μm)*. Telorchis assula* (Dujardin, 1845) has a tapering caudal end, testes contiguous, and a large cirrus sac (42). In *Telorchis parvus* Braun, 1901 the intestines terminate anteriorly to the testis (43).

#### 3.1.2 Monogenea

**Figure 2:**
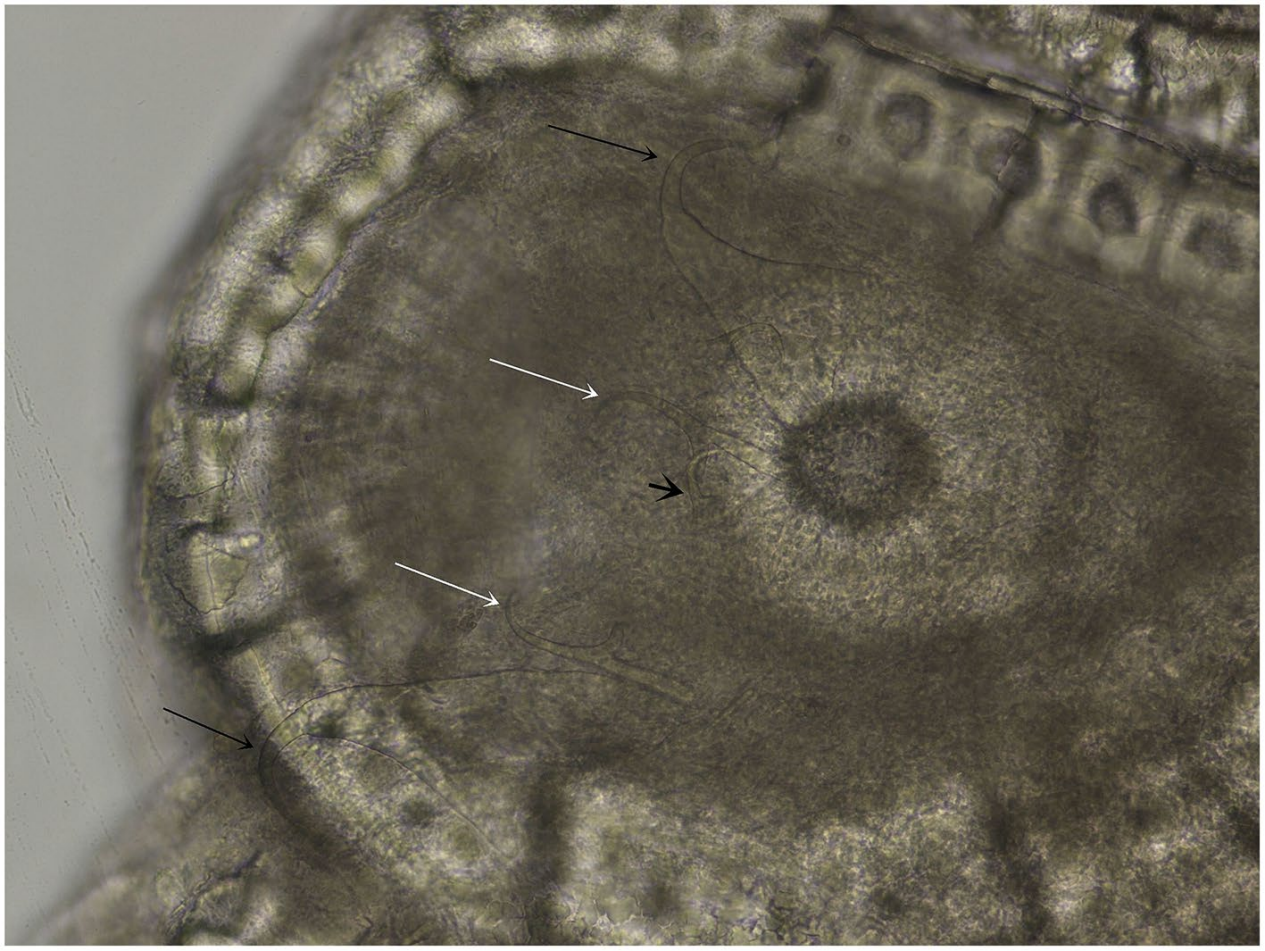
Detail of haptoral hamuli of *Polystomoides oris* isolated from the stomach of *Trachemys scripta*. Black arrow: larger hamulus; white arrow: smaller hamulus; short arrow: marginal hooklet. Bar = 50 μm.

Overall, 11 monogenean specimens were detected in the stomachs of 4 animals, presumably having migrated postmortem from the pharyngeal region. Host suborder, i.e., Cryptodira, and micro-habitat tropism, along with the morphology - elongated body, tapering anteriorly with a posterior haptor showing six suckers and marginal hooklets, median genital pore - permitted to ascribe them to the family Polystomatidae. Presence of two pairs of hamuli, smaller than the haptoral suckers, with the outer pair being larger, allowed to incorporate the specimens to the genus *Polystomoides* Ward, 1917 (Figure 2). Discriminant features for the species were as follows: the ratio between testis length and width ranged between 1.1 and 1.2; on the genital bulb, 28 to 33 genital spines were observed. Furthermore, the shape of the outer hamuli was similar to *P. pauli* Timmers and Lewis 1979 (44), with the large hooks showing entire, not bifid, roots. Further morphometrical details are given in Table 4.

**Table 4:** Morphometric parameters (μm) of 7 specimens of *Polystomoides oris*, collected from the stomach of *Trachemys scripta* (*n =* 4).

Morphometric characteristic	Min-max (*n*) (mean; DS)
Body Length	2,291–5,134 (6) (3,364; 1,070)
Body Width	411–999 (5) (662; 287)
Oral Sucker width	194
Haptoral sucker diameter	303–374 (4) (367; 32)
Haptoral L/BL	0.3–0.4 (2) (0.27; 0.09)
Haptor length	554–1,300 (3) (907; 375)
Haptor width	507–1,352 (3) (967; 427)
Hamulus total length	88 (1)
Hamulus handle length	79 (1)
Hamulus guard length	65 (1)
Hamulus hook length	30 (1)
Marginal hooklet length	25 (2)
Pharynx length	237–467 (3) (412; 115)
Pharynx width	241–634 (3) (570; 201)
Testis Length	371–376 (2) (373; 3.5)
Testis Width	307–321 (2) (314; 9.9)
Testis L/W	1.16–1.22 (2) (1.19; 0.05)
Ovary Length	181 (1)
Ovary Width	250 (1)
Genital bulb position	19–22 (2) (21; 2.14)
Genital bulb length	123–217 (4) (180; 45)
Genital spine no.	28–33
Genital spine length	40–45 (2) (43; 3.3)
Eggs	279×229-196 (2) (279 × 212)

n, sample size; L, length; W, width; DS, standard deviation.

*Remarks*: Eighteen species are reported within the genus *Polystomoides* showing hamuli on the haptor, all of which, with exception of *P. albicollis* (MacCallum, 1918), occurring in the oral cavity of the host. Among these, five species are reported to have a number of genital spines comparable to that of our samples, i.e., *Polystomoides coronatus* (Leidy, 1888) (n. 32), *P. cyclemydis* (Fischthal and Kuntz, 1964) (n. 32), *P. microcotyle* (Stunkard 1916) (n.32), *P. ocellatus* (Rudolphi, 1819) (n. 29–34), *P. rhodei* (Mañe-Garzón and Holcman-Spector, 1968) (n. 29–32), the last being different from *P. coronatus* only for the size of pharynx and of genital spines (45). With the exception of *P. cyclemydis*, all other species (*P. coronatus*, *P. rohdei*, *P. microcotyle* and *P. ocellatus*) have a rounded testis. *P. rohdei* differs from our sample in the shape of the hamuli, which have a much more developed root and a well-defined handle. *Polystomoides microcotyle* only has one morphological description by Stunkard (41) and has been only reported in *Chrysemys picta* in North America. The species is regarded as possibly synonymous of *P. pauli* and *P. oris*. Finally, in *P. ocellatus* the hamuli also show a different shape from our sample, as illustrated by Knopffler and Combes (46), with large hamuli having a straight to convex base and minor hamuli with a different handle shape. Hamuli of our samples look indeed identical to those of *P. pauli*, but this species differs in the number of genital spines (n. 36–49). By using the discriminant morphological characters suggested by Héritier et al. (31), with the addition of hamuli shape, we retain that reliable species identification of our isolates is not reachable. Hamuli of our samples look indeed identical to those of *P. pauli*, but this species differs in the number of genital spines (n. 36–49).

A total of 46 polystomatid monogeneans were isolated from the urinary bladders of 9 turtles and assigned to the family Polystomatidae based on the morphological criteria mentioned above. Based on host suborder and micro-habitat tropism, they were assigned to the genus *Polystomoides*. No hamuli were observed on the haptor. Sixteen genital spines were observed on the genital bulb; the testis shape was oval (mean length to width ratio: 1.46) (Table 5).

**Table 5:** Morphometric parameters (μm) of 16 specimens (15 adult and 1 immature) of *Polystomoides orbicularis*, collected from the urinary bladder of *Trachemys scripta* (*n =* 8).

Morphometric characteristic	Min-max (*n*) (mean; DS)
Body Length (BL)	1,610–4,250 (6) (2,869; 917)
Body Width (BW)	398–1,000 (5) (720; 248)
Oral Sucker width	270 (1)
Haptoral sucker diameter	93–273 (14) (218; 67)
Haptoral L/BL	0.21–0.27 (3) (0.26; 0.03)
Haptor length	515–1,048 (5) (700; 204)
Haptor width	543–1,018 (5) (738; 184)
Pharynx length	182–410 (3) (266; 391)
Pharynx width	251–313 (3) (280; 311)
Testis Length	270–358 (4) (324; 38)
Testis Width	192–251 (4) (223; 25)
Testis L/W	1.23–1.74 (4) (1.46)
Ovary Length	97–171 (2) (134)
Ovary Width	115–132 (2) (123)
Ovary-anterior tip distance	28–33 (2) (30)
Genital bulb position*	7–23 (3) (15; 8)
Genital bulb width	91–196 (5) (130; 46)
Genital spine no.	16
Genital spine length	31–54 (4) (42; 9)
Eggs	238-338×176-292 (4) (280×233; 42–48)

n, sample size; L, length; W, width; DS, standard deviation. *Distance from anterior end as percentage of body length.

*Remarks*: Twenty-eight species are reported within the genus *Polystomoides* (45); eleven out of them, formerly reported as belonging to genus *Neopolystoma* Price, 1939, show no hamuli on the haptor, i.e., *P. aspidonectis* (MacCallum, 1919), *P. cayensis* (Du Preez, Badets, Héritier and Verneau, 2017), *P. cyclovitellum* (Caballero, Zerecero and Grocott, 1957), *P. digitatus* (McCallum, 1918), *P. domitilae* (Caballero, 1938), *P. euzeti* (Combes and Ktari, 1976), *P. exhamatum* Ozaki, 1935, *P. opacus* (Du Preez, Landman and Verneau, 2023), *P. orbicularis* (Stunkard, 1916), *P. rugosus* (McCallum 1918) and *P. terrapenis* (Harwood, 1932), here including species reported from Nearctic and Palearctic regions. Among these, seven are reported in the urinary bladder of several Cryptodira species (45), with different host species-specificity. Considering the number of genital spines as a first morphological marker for species identification (31), five of them are reported to have 16–18 spines bordering genital bulb, i.e., *P. cayensis*, *P. cyclovitellum*, *P. exhamatum*, *P. orbicularis* and *P. terrapenis*. Among these, *P. cayensis* has a larger testis with different morphology compared to our specimens, and *P. exhamatum* has much larger body size. Three species are therefore comparable with our material in terms of microhabitat and morphology, i.e., *P. orbicularis*, *P. cyclovitellum* and *P. terrapenis*.

Other morphological features of *P. cyclovitellum*, such as a larger body size and different genital spine morphology, as reported by Du Preez et al. (45), are not compatible with our specimens. Moreover, there is only one record for this species from *Rhinoclemmys melanosterna* from Panama, Central America. *Polystomoides terrapenis* is regarded as very similar to *P. orbicularis* by Price (47), who also suggested that the two species may be identical; nevertheless, this species is only reported from *Terrapene carolina*, in Northern America. Therefore, our isolates were identified as *P. orbicularis*. The type host species for *P. orbicularis* corresponds to ours, and geographical reports match with the introduction of the species into the Palearctic from North America (18, 20).

#### 3.1.3 Nematoda

**Figure 3:**
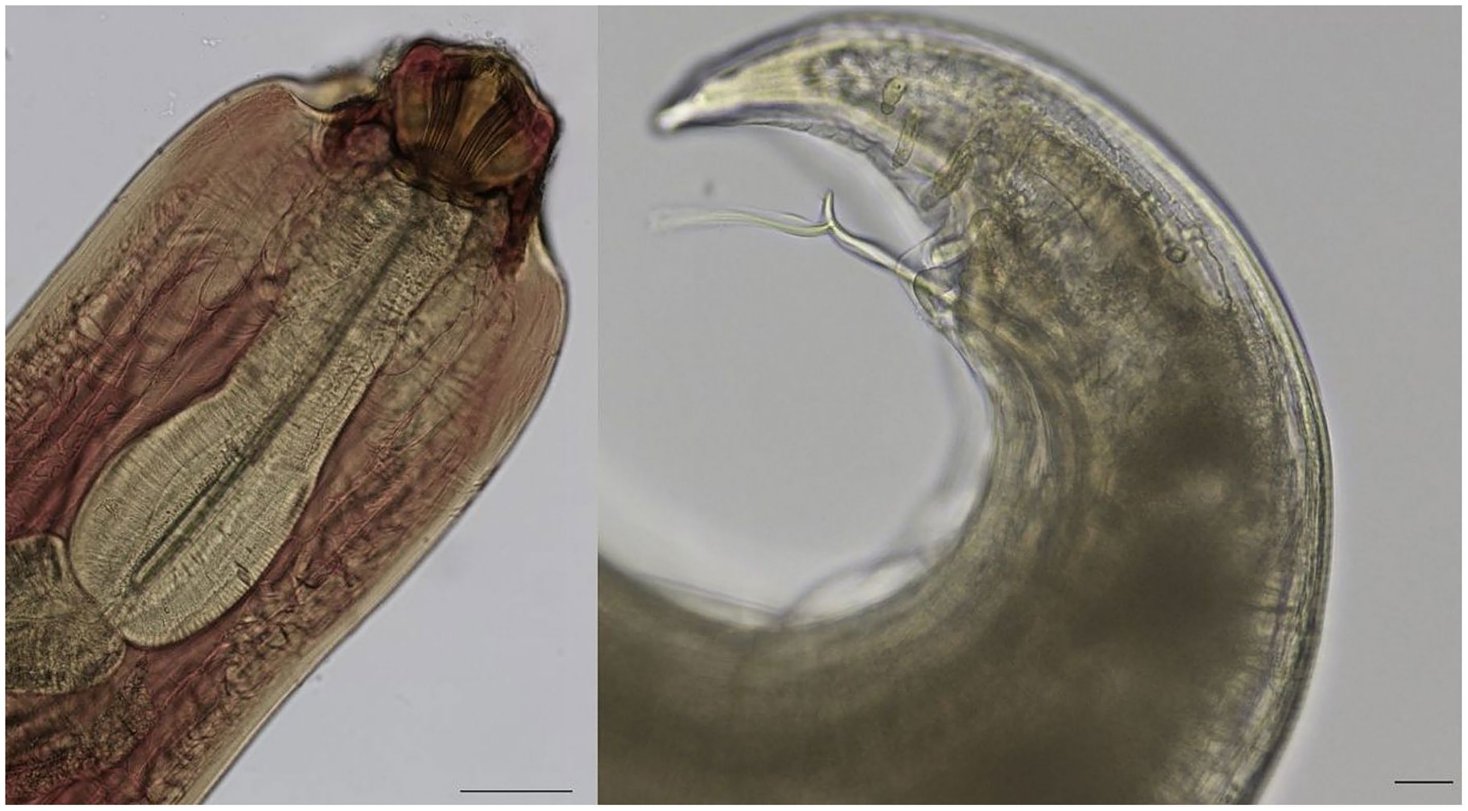
*Serpinema microcephalus* isolated from the intestine of *Trachemys scripta:* **(A)** Cephalic end, showing the buccal capsule with 8 ridges, and muscular esophagus. Bar = 200 μm; **(B)** detail of male caudal end showing the diverging process on the left spicule; three pairs of post anal papillae are also visible. Bar = 20 μm.

A total of 72 nematodes were recovered from 13 turtles, which were attributed to the genus *Serpinema* Yeh 1960 (Spirurida: Camallanidae) due to the presence of two lateral valves of the buccal capsule marked by complete longitudinal ridges clearly divided into dorsal and ventral groups (48, 49). The body is translucent orange, fusiform, and ranges in size from 7.4 to 9.8 mm (Table 6; Figure 3). The nematodes were identified as *Serpinema microcephalus* (Dujardin 1845) based on the following morphologic characteristics: cephalic structures included 8 thick ridges on buccal valves, well divided between ventral and dorsal by a median zone devoid of ridges. The buccal capsule is laterally compressed and composed of two valves together with a basal ring. The anterior margins of the valves are each edged by a sclerotised plate. Each valve is supported by a trident, consisting of three posteriorly directed prongs. The muscular esophagus is well developed, markedly separated from the glandular portion, and the latter is well developed and slender. *Female*: uterus filled with larvae; conical caudal tip, with three spike-like cuticular processes at the extremity. *Male*: caudal papillae divided into pre-anal (*n =* 7), and post anal (*n =* 6); among the latter, two pairs are located close to the anus and one even closer to the extremity; two unequal spicules, with the right one much more visible, well developed (range 689–809) and with a diverging process starting at 59 from the end.

**Table 6:** Morphometric parameters (μm) of 15 specimens (8 females and 7 males) of *Serpinema microcephalus*, collected from the intestine of *Trachemys scripta* (*n =* 7).

Morphometric characteristic	Female♀	Male♂
	Min-max (*n*) (mean; DS)	Min-max (*n*) (mean; DS)
Body Length	7,994–9,825 (3) (8,839; 924)	7,482–11,165 (4) (8,882; 4,217)
Body Width	253–513 (6) (354; 95)	244–288 (3) (267; 22)
Basal ring Width	104 (2) (104)	69–310 (3) (177; 126)
Number of ridges	8 (8)	8 (7)
Pair of male caudal papillae	–	7 preanal, 6 post anal
Muscular esophagus length	239–442 (4) (343; 110)	352–391 (4) (377; 18)
Glandular esophagus length	402–506 (3) (448; 53)	580–697 (4) (648; 50)
Tail	139–222 (3) (183; 173)	–
Caudal alae	–	Present
Right spicule Length	–	689–809 (3) (722; 62)
Diverging process to spicule tip distance	–	59 (1)

n, sample size; DS, standard deviation.

*Remarks*: The genus *Serpinema* Yeh, 1960 includes parasites of freshwater turtles in the Oriental, Neotropical, and Palearctic regions. Baker (50) reported *Serpinema trispinosum* (Leidy, 1852) as the most widespread species of its genus in emydid turtles in Northern America, parasitizing *T. scripta*, and is the only species reported in this host. In Europe, only *S. microcephalus* has been reported, witini the hosts *M. leprosa* and *E. orbicularis* (51–53). Morphological differentiation relies on the *S. trispinosum* valves, which are relatively wider and shorter, and ridges in each buccal valve vary from 15 to 19. In *S. microcephalus* ridges are less numerous (8–11) but thicker (50).

### 3.2 Copromicroscopic examination

Forty-two individual fecal samples could be analyzed due to a lack of fecal material in the rectum of the remaining 20 turtles. Fifteen fecal samples were found to be positive for at least one parasitic genus. Brown oval eggs, measuring 29–35 × 17–20 μm (27 ± 6.8 × 17 ± 0.5), identical to those observed in the uterus of the *T. corti*, were observed in 9 animals. All animals except one were found positive for *T. corti* at the intestinal level. Eggs referable to the family Capillaridae were detected in the feces of 2 animals, measuring 62–66 × 28–32 μm (65.0 ± 2 × 30 ± 2), having rough surface and two, non-protruding, bipolar plugs.

### 3.3 Molecular biology

Four consensus sequences of 28S rDNA gene were obtained from four digenean trematodes. Their comparison showed complete identity between isolates, and Blast analysis revealed 99.5% identity with *Telorchis corti* (acc. Number MK648312) along their alignment (1,270 bp). Amplification and sequencing of the COX1 gene yielded 16 consensus sequences, each from a different host, with few single nucleotide polimorphisms (10/330 bp), and the highest identity (97–99.3%) with *Telorchis* sp. HS82 (acc. Number LC818892) across their alignment (330 bp). The newly generated sequences were deposited in GenBank (acc. Numbers PZ093117-120 for 28S, PZ094492-507 for COX1).

From monogeneans isolated from the urinary bladder, amplification and sequencing of 28S rDNA gene yielded 2 high-quality consensus sequences (acc. Numbers PZ113709-710) and their alignment (1,300 bp long) showed they were identical. The Blast research in GenBank revealed them to be highly similar (99.8%) to the sequence of *Polystomoides orbicularis* (syn. *Neopolystoma orbicularis*) (acc. Number MW219725) along their alignment (1,290 bp). No sequences of 28S were generated from monogeneans from the stomach.

Only one sequence of COX1 gene (390 bp) could be obtained from a monogenean from the urinary bladder of one individual (acc. Number PZ094665). The sequence appeared to have the highest identity with *Polystomoides orbicularis* (syn. *Neopolystoma orbicularis*) (acc. Number KY704697) (98.9%) along their alignment (381 bp) at the Blast search in GenBank. Three sequences of COX1 gene were retrieved from monogeneans isolated from the stomach of 2 individuals (acc. Numbers PZ094569-571), and their alignment proved them to be identical (370 bp). The Blast research in GenBank revealed high identity (99.7%) with *Polystomoides oris* (acc. Number KY704842) along their alignment (348 bp).

Two consensus sequences were obtained for 28S gene from nematodes (acc. Numbers PZ113711-712). Their alignment (1,200 bp) proved them to be identical. The Blast research in GenBank reported the highest similarity with sequences of *Serpinema* sp. (acc. Number MZ056548) (95.1%) along their alignment (1,060 bp). No sequences of COX1 gene were obtained.

### 3.4 Histopathology

**Figure 4:**
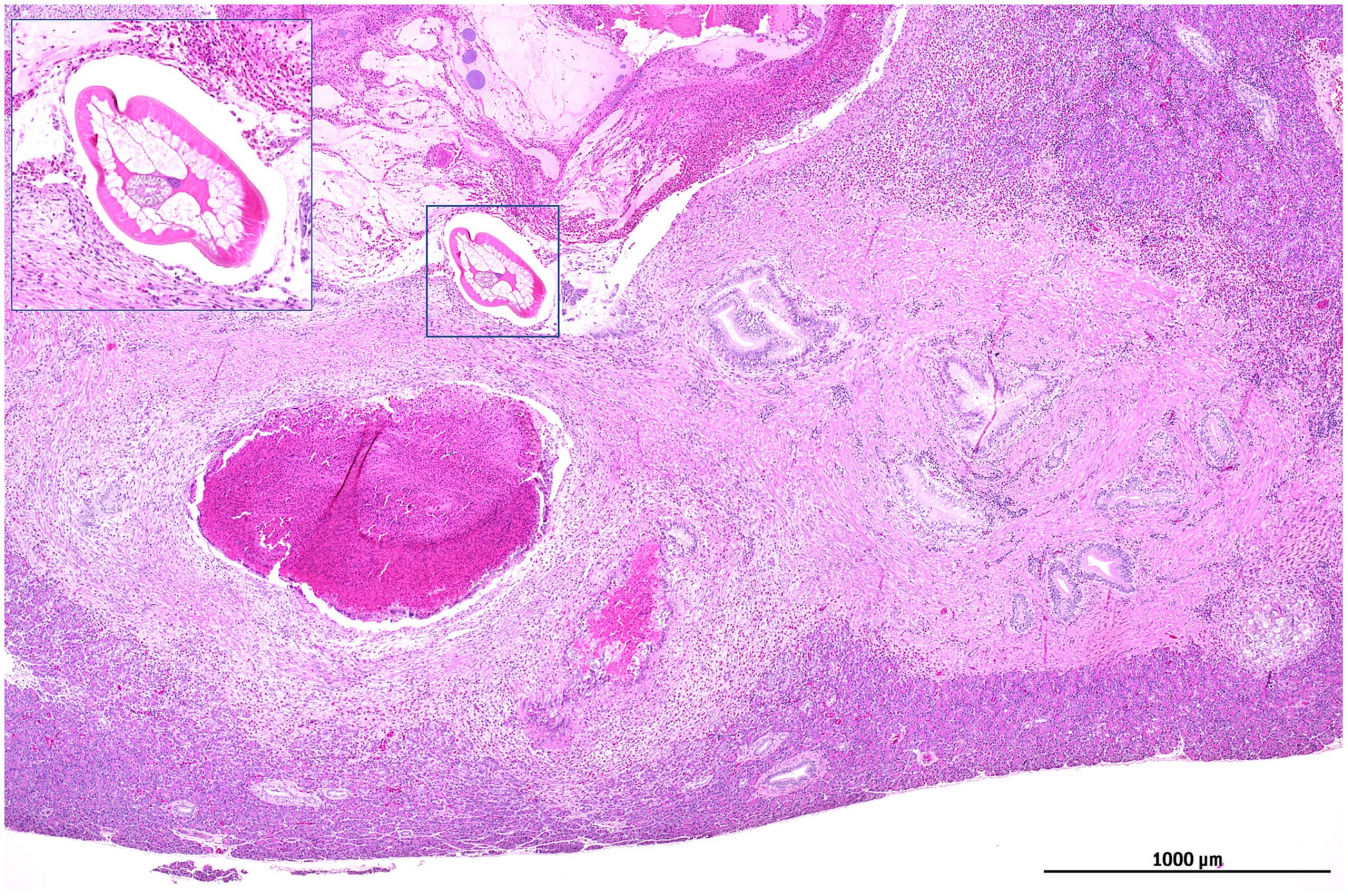
*Trachemys scripta*, pancreas. Well-organized granulomas with central necrosis encircled by an extensive mixed inflammatory infiltrate with prominent peripheral fibrosis. A cross-section of a parasite at the periphery of the granuloma (square) can be seen, attributable to a nematode; its high magnification is visible in the square inset.

Necropsy of four turtles revealed consistent, grossly visible nodular lesions within the pancreatic parenchyma, characterized by either multifocal distribution or coalescent masses. Histopathological analysis demonstrated well-organized granulomas with central caseous necrosis encircled by an extensive mixed inflammatory infiltrate comprising lymphocytes, eosinophils, and Langhans-type multinucleated giant cells, demarcated by prominent peripheral fibrosis. Within one specimen, a near-transverse histological section of a metazoan parasite was identified at the granuloma periphery. Morphological characteristics, including a relatively thick cuticle, coelomyarian musculature, and a simple tubular intestine occupying the pseudocoelomic cavity, were consistent with nematode identification (Figure 4).

## 4 Discussion

Estimates of red eared sliders (*Trachemys scripta*) introduced in Europe number in the millions (54), and the species is present throughout Italy, with higher abundance in the Northern and central regions (55). Although several studies have investigated its role as reservoir and vector of zoonotic diseases in the country (56–58), no research has yet addressed its potential impact on the health of other animal species. In this survey, we report the co-introduction with *T. scripta* of three species of helminths which are traceable to its native geographical range, namely *Telorchis corti*, *Polystomoides orbicularis* and *Polystomoides oris*. Furthermore, spillover of the nematode *Serpinema microcephalus*, which is endemic to European freshwater turtles, is reported in this invasive host species.

Trematodes of the genus *Telorchis* (Digenea: Telorchiidae) are heteroxenous intestinal parasites of freshwater turtles, snakes and salamanders. Taxonomy of this genus has been the subject of repeated controversy (59) and the lack of deposited molecular data limits the possibility of resolving taxonomic issues. Preliminary molecular data support our morphological identification of *T. corti*, which is here reported for the first time in Europe. A broad host spectrum has been described for this trematode, spanning across the Reptilia, including *T. scripta* and nine other genera of Emydidae (60, 61). Given the generalist nature of this species, a spillover event to sympatric population of *E. orbicularis* could be expected, potentially adding this species to the long list of reported hosts for *T. corti*.

Monoxenous parasites of the family Polystomatidae mainly infect amphibians and freshwater turtles. Species reported from chelonians, infecting either the urinary bladder, the conjunctival sac or oral mucosa, are divided into nine accepted genera: *Polystomoides*, *Polystomoidella*, *Apaloneotrema*, *Aussietrema*, *Fornixtrema*, *Manotrema*, *Pleurodirotrema*, *Uteropolystomoides*, and *Uropolystomoides* (62). Following recent revision, the genus *Polystomoides* now includes all polystomatids infecting the urinary bladder or oral cavity of cryptodiran turtles (62). In this study, the use of mtCOX1 analysis proved essential to help species identification in the case of *P. oris* (18). Observation of discriminant morphological characters intra-genus, i.e., the number of genital spines and testis shape (31), was not sufficient for species identification in our case. The variability in the number of genital spines within each species, and its overlap across different species sharing the same microhabitat, complicates the identification, making molecular data invaluable for species determination. In the light of molecular results and morphometric observation, we consider synonymy of *P. oris* and *P. pauli* as potentially valid as suggested by Du Preez et al. (45).

Monogeneans of the family Polystomatidae have been extensively studied in Southern Europe to explore parasite diversity and host-switching between American and European emydid turtles. Both species of polystomatids isolated in our study have been previously reported in both *T. scripta* and in the indigenous species *E. orbicularis* and *M. leprosa* in Southern Europe (18–20, 22). Héritier et al. (18) carried out the most comprehensive survey on polystomatids from both indigenous and exotic turtle species in Spain, France and North Africa. Eleven species of polystomatids were reported overall, eight of which were considered exotic. All species except one were isolated from *E. orbicularis*, including the two species isolated in our study. The simple life cycle of these monoxenous parasites, and the generalist nature of the two species *P. orbicularis* and *P. oris*, likely account for the co-invasion of these parasites with their host in the new environment. Low pathogenicity has been reported for *P. orbicularis* in *T. scripta,* although polypi and inflammation of the urinary bladder wall have been occasionally reported (63). The presence, abundance and pathology of the species should be investigated in *E. orbicularis*, without underestimating the potential enhanced virulence of this exotic polystomatid species in the naïve host.

Nematodes of the family Camallanidae Railliet and Henry, 1915 are parasites of the gastrointestinal tract of amphibians, fish, and reptiles (64). Twelve species are considered valid in the genus *Serpinema*, commonly identified by the morphology of their buccal capsule (64). One species is reported from *T. scripta* in North America, namely *Serpinema trispinosum*. Comparison with our sample allowed identification of the species *S. microcephalus*, which is historically reported in the Palearctic region in native turtle species (28). Low identity was retrieved from the alignment of our sequences with *Serpinema* spp. in GenBank, likely due to the absence of sequences of *S. microcephalus* in the database. Spillover of this Palearctic species to *T. scripta* has been previously reported in southwestern Spain (28), in eastern Spain (20, 29, 30) and in Japan (65) with variable prevalence. In the Spanish surveys, the presence of *S. microcephalus* in *T. scripta* has been associated to both intestinal and pancreatic lesions. Hard, necrotic nodules in the pancreas parenchyma, grossly and microscopically similar to ours, are described as consequence of *S. microcephalus* migration in pancreatic ducts of *T. scripta* in Spain (29). Martìnez-Silvestre et al. (30) also reported catarrhal, haemorrhagic to ulcerative enteritis in *Graptemys*, *Pseudemys*, *Mauremys* (syn. *Ocadia*) and *Trachemys*. On the other hand, the Nearctic species *S. trispinosum* is not associated to pathology in red eared sliders in their native range (66). Wieczorowski (67) reported large nodular pancreatic lesions (1–3 mm) in *T. scripta* (syn. *Chrysemis scripta*) with concomitant infection by *S. trispinosum* (syn *Camallanus trispinosus*) and *Telorchis nematoides*, but ascribed the lesions to the migration of the trematode *T. nematoides*. In our study, three out of four cases of pancreatic nodules were concomitant with simple intestinal infection by *S. microcephalus.* Although we cannot exclude past infections by other parasites, none of the turtles was infected by trematodes at the time of necropsy. Some other examples of increased virulence exist for species of Camallanidae in newly acquired host species (6, 8, 68). The highly successful invasive species *Camallanus cotti*, for example, is associated to severe hemorrhagic enteritis, with frequent fatal outcome, in tropical fish worldwide (69, 70). Our finding add further evidence to the hypothesis that exposure of naïve hosts to a new parasite species can result in more severe pathology than in the typical host.

One final remark concerns the finding of eggs referable to Capillariidae in fecal samples from two turtles. While several species of *Capillaria sensu lato* are reported from snakes (71), *Capillaria serpentina* Harwood 1932 is, to our knowledge, the only species described in turtles, namely from *Chelydra serpentina*, *Sternotherus odoratus* and the emydid *Chrysemys picta* in North America (72). Although pseudoparasitism cannot be completely ruled out, the very tiny size of the nematodes and their localization beneath the intestinal mucosal layer, may account for the absence of adult capillariid nematodes in the intestinal washes. Further investigations, aimed at isolation and description of adult parasites are warranted to elucidate the true host spectrum of the Capillariidae in reptiles.

Species richness was much lower compared to the updated literature on *T. scripta* from its native range (73, 74). Eleven helminth species are described in *T. scripta* from Oklahoma, encompassing three phyla and including the blood flukes *Spirorchis* sp. and *Diarmostorchis blandingii*, and the highly prevalent nematodes *Camallanus* sp. and *Spiroxys* sp. Easley (74) reports a similar richness, but includes three acanthocephalan and five nematode species. Species richness is also lower in *T. scripta* in other non-native areas. Three helminth species are reported in populations from western Spain (20), and similarly, Hidalgo-Vila et al. (28) described only four nematode species composing the intestinal helminth community. Finally, Cardells et al. (75) report *Telorchis attenuata* in eastern Spain as the only parasite species in *T. scripta*, found with low prevalence (7.6%). The so called enemy release hypothesis, whereby alien species lose part of their native parasitofauna following introduction to a new area, may account for this disparity in parasite richness. Enhanced pathology caused by the locally native species *S. microcephalus* may counterbalance this favorable condition by affecting the fitness of the population. Nevertheless, spillback effects on *E. orbicularis* should not be ruled out, as the presence of a new, competent host may increase the abundance of infective stages in the environment, possibly altering disease dynamics.

## 5 Conclusion

This study on the helminths harbored by the invasive species *T. scripta* is the first conducted in Italy, and offers an initial insight into co-introduced parasite species. Three exotic helminth species are reported, all being generalist species capable of infecting several hosts in the family Emydidae. The effects of newly introduced parasite species on naïve native turtles remain to be elucidated. Further research on the parasitofauna of *E. orbicularis* is strongly recommended, particularly in populations exhibiting varying degrees of sympatry with the exotic species *T. scripta*. Such studies would help evaluate potential parasite-mediated detrimental effects of the invasive red-eared slider on the population fitness of native freshwater turtles. Finally, a broader survey is advised across Italy to examine red eared slider populations in other regions and natural contexts, to uncover any additional phenomena of co-introduction of exotic helminths.

## Data Availability

The sequences generated for this study can be found in the GenBank with Accession Number: PZ093117 - PZ093120 (*Telorchis corti*, 28S); PZ113709- PZ113710 (*Polystomoides orbicularis*, 28S); PZ113711- PZ113712 (*Serpinema microcephalus*, 28S); PZ094492- PZ094507 (*Telorchis corti*, COX1); PZ094569- PZ094571 (*Polystomoides oris*, COX1); PZ094665 (*Polystomoides orbicularis*, COX1).

## References

[1] Seebens H Meyerson LA Richardson DM Lenzner B Tricarico E Courchamp F . Biological invasions: a global assessment of geographic distributions, long‐term trends, and data gaps. Biol Rev. (2025) 100:2542–83. doi: 10.1111/brv.70058, 40793987 PMC12586311

[2] Vitousek PM d’Antonio CM Loope LL Westbrooks R. Biological invasions as global environmental change. Am Sci. (1996) 84:468–78.

[3] Linders TEW Schaffner U Eschen R Abebe A Choge SK Nigatu L . Direct and indirect effects of invasive species: biodiversity loss is a major mechanism by which an invasive tree affects ecosystem functioning. J Ecol. (2019) 107:2660–72. doi: 10.1111/1365-2745.13268

[4] Lodge DM. Biological invasions: lessons for ecology. Trends Ecol Evol. (1993) 8:133–7. doi: 10.1016/0169-5347(93)90025-K, 21236129

[5] Havel JE Kovalenko KE Thomaz SM Amalfitano S Kats LB. Aquatic invasive species: challenges for the future. Hydrobiologia. (2015) 750:147–70. doi: 10.1007/s10750-014-2166-0, 32214452 PMC7087615

[6] Lymbery AJ Morine M Kanani HG Beatty SJ Morgan DL. Co-invaders: the effects of alien parasites on native hosts. Int J Parasitol Parasites Wildl. (2014) 3:171–7. doi: 10.1016/j.ijppaw.2014.04.002, 25180161 PMC4145144

[7] Taraschewski H. Hosts and parasites as aliens. J Helminthol. (2006) 80:99–128. doi: 10.1079/JOH2006364, 16768855

[8] Prenter J MacNeil C Dick JTA Dunn AM. Roles of parasites in animal invasions. Trends Ecol Evol. (2004) 19:385–90. doi: 10.1016/j.tree.2004.05.002, 16701290

[9] Kelly DW Paterson RA Townsend CR Poulin R Tompkins DM. Parasite spillback: a neglected concept in invasion ecology? Ecology. (2009) 90:2047–56. doi: 10.1890/08-1085.1, 19739367

[10] Chalkowski K Lepczyk CA Zohdy S. Parasite ecology of invasive species: conceptual framework and new hypotheses. Trends Parasitol. (2018) 34:655–63. doi: 10.1016/j.pt.2018.05.008, 29935995

[11] Haubrock PJ Everts T Abreo NAS Bojko J Deklerck V Dickey JWE . The impacts of biological invasions. Biol Rev. (2025) 22:70124. doi: 10.1002/brv.70124 PMC13149820 41467425

[12] Dunn AM. Parasites and biological invasions. Adv Parasitol. (2009) 9:161–84. 10.1016/S0065-308X(08)00607-6 19289194

[13] Ficetola GF Padoa-Schioppa E Monti A Massa R Bernardi FD Bottoni L. The importance of aquatic and terrestrial habitat for the European pond turtle (*Emys orbicularis*): implications for conservation planning and management. Can J Zool. (2004) 82:1704–12. doi: 10.1139/z04-170

[14] Fritz U Chiari Y. Conservation actions for European pond turtles – a summary of current efforts in distinct European countries. Herpetol. Notes. (2013) 6:105.

[15] Cadi A Joly P. Competition for basking places between the endangered European pond turtle (*Emys orbicularisgalloitalica*) and the introduced red-eared slider (*Trachemys scripta elegans*). Can J Zool. (2003) 81:1392–8. doi: 10.1139/z03-108

[16] Cadi A Joly P. Impact of the introduction of the red-eared slider (*Trachemys scripta elegans*) on survival rates of the European pond turtle (*Emys orbicularis*). Biodivers Conserv. (2004) 13:2511–8. doi: 10.1023/B:BIOC.0000048451.07820.9c

[17] Schönbächler K Olias P Richard OK Origgi FC Dervas E Hoby S . Fatal spirorchiidosis in European pond turtles (*Emys orbicularis*) in Switzerland. Int J Parasitol Parasites Wildl. (2022) 17:144–51. doi: 10.1016/j.ijppaw.2022.01.004, 35079570 PMC8777241

[18] Héritier L Valdeón A Sadaoui A Gendre T Ficheux S Bouamer S . Introduction and invasion of the red-eared slider and its parasites in freshwater ecosystems of southern Europe: risk assessment for the European pond turtle in wild environments. Biodivers Conserv. (2017) 26:1817–43. doi: 10.1007/s10531-017-1331-y

[19] Meyer L Du Preez L Bonneau E Héritier L Quintana M Valdeón A . Parasite host-switching from the invasive American red-eared slider, *Trachemys scripta elegans*, to the native Mediterranean pond turtle, *Mauremys leprosa*, in natural environments. Aquat Invasions. (2015) 10:79–91. doi: 10.3391/ai.2015.10.1.08

[20] Domènech F Marquina R Soler L Valls L Aznar FJ Fernández M . Helminth fauna of the invasive American red-eared slider *Trachemys scripta* in eastern Spain: potential implications for the conservation of native terrapins. J Nat Hist. (2016) 50:467–81. doi: 10.1080/00222933.2015.1062931

[21] Iglesias R García-Estévez J Ayres C Acuña A Cordero-Rivera A. First reported outbreak of severe spirorchiidiasis in *Emys orbicularis*, probably resulting from a parasite spillover event. Dis Aquat Org. (2015) 113:75–80. doi: 10.3354/dao02812, 25667339

[22] Verneau O Palacios C Platt T Alday M Billard E Allienne JF . Invasive species threat: parasite phylogenetics reveals patterns and processes of host-switching between non-native and native captive freshwater turtles. Parasitology. (2011) 138:1778–92. doi: 10.1017/S0031182011000333, 21767431

[23] Sancho Alcayde V Lacomba Andueza JL Bataller Gimeno JV Pradillo Carrasco A. Manual para el Control y Erradicación de Galápagos Invasores. Colección Manuales Técnicos de Biodiversidad. Valencia: Conselleria d’Agricultura. Medi Ambient Canvi Climàtic Desenvol Rural General Valencia Valencia Spain (2015). p. 78.

[24] Smith KG Nunes AL Aegerter J Baker SE Di Silvestre I Ferreira CC . A Manual for the Management of Vertebrate Invasive alien Species of Union Concern, Incorporating animal Welfare. Technical Report Prepared for the European Commission within the Framework of the contract no. 07.027746/2019/812504/SER/ENV.D.2. Brussels: European Commission (2022).

[25] Shilton C. "Necropsy". In: Donely B Monks D Johnson R Brendan C, editors, 1a Edn. Hoboken: Wiley Blackwell (2018)

[26] Mitchell JC Pague CA. Body size, reproductive variation, and growth in the slider turtle at the northeastern edge of its range. Life Hist Ecol Slider Turt Smithson Inst. Washington, DC, USA: Smithsonian Institution Press. (1990) 22:146–51.

[27] Marchiori E Negrisolo E Cassini R Garofalo L Poppi L Tessarin C . Cardiovascular flukes (Trematoda: Spirorchiidae) in *Caretta caretta* Linnaeus, 1758 from the Mediterranean Sea. Parasit Vectors. (2017) 10:467. doi: 10.1186/s13071-017-2396-x, 29017541 PMC5633879

[28] Hidalgo-Vila J Díaz-Paniagua C Ribas A Florencio M Pérez-Santigosa N Casanova JC. Helminth communities of the exotic introduced turtle, *Trachemys scripta elegans* in southwestern Spain: transmission from native turtles. Res Vet Sci. (2009) 86:463–5. doi: 10.1016/j.rvsc.2008.08.003, 18799176

[29] Hidalgo-Vila J Martiínez-Silvestre A Ribas A Casanova JC Pérez-Santigosa N Díaz-Paniagua C. Pancreatitis associated with the helminth *Serpinema microcephalus* (Nematoda: Camallanidae) in exotic red-eared slider turtles (*Trachemys scripta elegans*). J Wildl Dis. (2011) 47:201–5. doi: 10.7589/0090-3558-47.1.201, 21270009

[30] Martínez-Silvestre A Guinea D Ferrer D Pantchev N. Parasitic enteritis associated with the camallanid nematode *Serpinema microcephalus* in wild invasive turtles (*Trachemys*, *Pseudemys*, *Graptemys*, and *Ocadia*) in Spain. J Herpetol Med Surg. (2015) 25:48–52. doi: 10.5818/1529-9651-25.1.48

[31] Héritier L Verneau O Smith KG Coetzer C Du Preez LH. Demonstrating the value and importance of combining DNA barcodes and discriminant morphological characters for polystome taxonomy (Platyhelminthes, Monogenea). Parasitol Int. (2018) 67:38–46. doi: 10.1016/j.parint.2017.03.002, 28336417

[32] Sharma RSK Rigby MC Sumita S Sani RA Vidyadaran MK Jasni S . Redescription of *Serpinema octorugatum* (Baylis, 1933) (Nematoda: Camallanidae) from the Malayan box turtle *Cuora amboinensis* (Daudin) (Chelonia: Bataguridae). Syst Parasitol. (2002) 53:19–28. doi: 10.1023/A:1019997922052, 12378130

[33] Bush AO Lafferty KD Lotz JM Shostak AW. Parasitology meets ecology on its own terms: Margolis et al. revisited. J Parasitol. (1997) 83:575. doi: 10.2307/3284227 9267395

[34] Reiczigel J Marozzi M Fábián I Rózsa L. Biostatistics for parasitologists–a primer to quantitative parasitology. Trends Parasitol. (2019) 35:277–81. doi: 10.1016/j.pt.2019.01.003, 30713051

[35] Olson PD Cribb TH Tkach VV Bray RA Littlewood DTJ. Phylogeny and classification of the Digenea (Platyhelminthes: Trematoda). Int J Parasitol. (2003) 33:733–55. doi: 10.1016/S0020-7519(03)00049-3, 12814653

[36] Folmer O Black M Hoeh W Lutz R Vrijenhoek R. DNA primers for amplification of mitochondrial cytochrome c oxidase subunit I from diverse metazoan invertebrates. Mol Mar Biol Biotechnol. (1994) 3:294–9. 7881515

[37] Littlewood DTJ Rohde K Clough KA. Parasite speciation within or between host species?—phylogenetic evidence from site-specific polystome monogeneans. Int J Parasitol. (1997) 27:1289–97. doi: 10.1016/S0020-7519(97)00086-6, 9421713

[38] Pauly A Schuster R Steuber S. Molecular characterization and differentiation of opisthorchiid trematodes of the species Opisthorchis felineus (Rivolta, 1884) and Metorchis bilis (Braun, 1790) using polymerase chain reaction. Parasitol Res. (2003) 90:409–14. doi: 10.1007/s00436-003-0851-4, 12748848

[39] Altschul SF Gish W Miller W Wmyers E Lipman DJ. Basic Ocal al gnment search tool. J Mol Biol. (1990) 215:403–10. 2231712 10.1016/S0022-2836(05)80360-2

[40] Brooks DR O’Grady RT Glen DR. Phylogenetic analysis of the Digenea (Platyhelminthes: Cercomeria) with comments on their adaptive radiation. Can J Zool. (1985) 63:411–43.

[41] Stunkard HW. Notes on the trematode genus *Telorchis* with descriptions of new species. J Parasitol. (1915) 2:57. doi: 10.2307/3271020

[42] Shayegh H Rajabloo M Gholamhosseini A Mootabi Alavi A Salarian P Zolfaghari A. Endohelminths of European pond turtle *Emys orbicularis* in Southwest Iran. J Parasit Dis. (2016) 40:194–8. doi: 10.1007/s12639-014-0477-8, 27065624 PMC4815831

[43] Stossich M. Alcuni distomi della collezione elmintologica del museo zoologico di Napoli. Naples: Melfi & Joele (1904).

[44] Timmers SF Lewis JPD. Helminths of *Chrysemys picta belli* in Manitoba including *Polystomoides pauli* sp. n.(Monogenea: Polystomatidae). Can J Zool. (1979) 57:1046–51.

[45] Du Preez LH Landman WJ Verneau O. Polystomatid Flatworms: State of Knowledge and Future Trends. Cham: Springer International Publishing (2023).

[46] Knoepffler LP Combes C. Présence en Corse de Polystomoides ocellatum (Rudolphi, 1819) chez *Emys orbicularis* (L., 1758) (Chelonia, Emydidae). Considérations sur la répartition mondiale du genre Polystomoides. Vie et Milieu. 27:221–30.

[47] Price EW. North American Monogenetic Trematodos. IV. The Family POLYSTOMATIDAE (Polystomatoidea). (1939). p. 80–92.

[48] Liang-Sheng Y. On a reconstruction of the genus Camallanus Railliet and Henry, 1915. J Helminthol. (1960) 34:117–24. doi: 10.1017/S0022149X00020435, 13787293

[49] Chabaud AG. "Spirurida: Camallanoidea, Dracunculoidea, Gnathostomatoidea, Physalopteroidea, Rictularoidea and Thelazioidea". In: Anderson RC Chabaud AG Willmott S, editors. Keys to the nematode Parasites of Vertebrates: Archival Volume. London: CABI Wallingford UK (2009). p. 334–60.

[50] Baker MR. *Serpinema* spp.(Nematoda: camallanidae) from turtles of North America and Europe. Can J Zool. (1979) 57:934–9. doi: 10.1139/z79-114

[51] Dujardin F. Histoire naturelle des helminthes ou vers intestinaux, vol. 1 England: Roret (1845).

[52] Gagno S. Parasitologie des Chéloniens Helminthes. Chelonii. (2006) 5:1–108.

[53] Sánchez Torres N Martín JE Roca Velasco V. Intestinal helminth parasitizing *Mauremys leprosa* (Chelonia: Bataguridae) from Extremadura (western Spain). Rev Esp Herpetol. (2005) 19:47–55.

[54] Ficetola GF Rödder D Padoa-Schioppa E. "*Trachemys scripta* (slider terrapin)". In: Francis RA Hardwick T, editors. Handb Glob Freshw Invasive Species. London: Routledge. (2012). p. 331–9.

[55] Tsiamis K Gervasini E Deriu I Cardoso A. Updates on the Baseline Distribution of Invasive Alien Species of Union Concern (2019). Luxembourg: Publications Office of the European Union (2019).

[56] Dezzutto D Barbero R Canale G Acutis PL Biolatti C Dogliero A . Detection of *Leptospira* spp. in water turtle (*Trachemys scripta*) living in ponds of urban parks. Vet Sci. (2017) 4:51. doi: 10.3390/vetsci4040051, 29056709 PMC5753631

[57] Bonacina E Oltolina M Robbiati R Pinzauti P Ebani VV. Serological survey on the occurrence of anti-*Leptospira* spp. antibodies in red-eared terrapins (*Trachemys scripta elegans*) living in a natural park of northern Italy. Animals. (2021) 11:602. doi: 10.3390/ani11030602, 33668811 PMC7996346

[58] Moroni B Meletiadis A Di Nicola MR Garcia-Vozmediano A Pitti M Dipietromaria G . Prevalence of *Salmonella*, *Cryptosporidium* and *Leptospira* in invasive pond slider (*Trachemys scripta*) in North-Western Italy. Vet Med Sci. (2025) 11:e70439. doi: 10.1002/vms3.70439, 40536911 PMC12178313

[59] Macdonald CA Brooks DR. Revision and phylogenetic analysis of the north American species of *Telorchis* Luehe, 1899 (Cercomeria: Trematoda: Digenea: Telorchiidae). Can J Zool. (1989) 67:2301–20. doi: 10.1139/z89-324

[60] Moravec F Vargas-Vázquez J. Some endohelminths from the freshwater turtle *Trachemys scripta* from Yucatan, Mexico. J Nat Hist. (1998) 32:455–68. doi: 10.1080/00222939800770241

[61] Radtke A McLennan DA Brooks DR. Resource tracking in north American *Telorchis* spp. Digenea: Plagiorchiformes: Telorchidae. 88:874–9. 10.1645/0022-3395(2002)088[0874:RTINAT]2.0.CO;2 12435123

[62] Chaabane A Du Preez L Johnston GR Verneau O. Revision of the systematics of the Polystomoidinae (Platyhelminthes, Monogenea, Polystomatidae) with redefinition of *Polystomoides* Ward, 1917 and *Uteropolystomoides* Tinsley, 2017. Parasite. (2022) 29:56. doi: 10.1051/parasite/2022056, 36562437 PMC9879127

[63] Henke SE Pence DB. Urinary bladders of freshwater turtles as a renal physiology model potentially biased by monogenean infections. Lab Anim Sci. (1990) 40:172–7. 2157098

[64] Moravec F. Nematodes of Freshwater Fishes of the neotropical region. Praha: Academia (1998).

[65] Oi M Araki J Matsumoto J Nogami S. Helminth fauna of a turtle species introduced in Japan, the red-eared slider turtle (*Trachemys scripta elegans*). Res Vet Sci. (2012) 93:826–30. doi: 10.1016/j.rvsc.2011.10.001 22047816

[66] Esch GW Gibbons JW Bourque JE. The distribution and abundance of enteric helminths in *Chrysemys* s. *scripta* from various habitats on the Savannah River Plant in South Carolina. J Parasitol. (1979) 65:624. doi: 10.2307/3280332

[67] Wieczorowski E. Parasitic lesions in turtles. J Parasitol. (1939) 25:395. doi: 10.2307/3272306

[68] Mastitsky SE Karatayev AY Burlakova LE Molloy DP. Biodiversity research: parasites of exotic species in invaded areas: does lower diversity mean lower epizootic impact? Divers Distrib. (2010) 16:798–803. doi: 10.1111/j.1472-4642.2010.00693.x

[69] Menezes RC Tortelly R Tortelly-Neto R Noronha D Pinto RM. *Camallanus cotti* Fujita, 1927 (Nematoda, Camallanoidea) in ornamental aquarium fishes: pathology and morphology. Mem Inst Oswaldo Cruz. (2006) 101:683–7. doi: 10.1590/s0074-02762006000600018 17072484

[70] Kim JH Hayward CJ Heo GJ. Nematode worm infections (*Camallanus cotti*, Camallanidae) in guppies (*Poecilia reticulata*) imported to Korea. Aquaculture. (2002) 205:231–5. doi: 10.1016/S0044-8486(01)00691-3

[71] Šlapeta J Modrý D Johnson R. "Reptile parasitology in health and disease". In: Doneley B Monks D Johnson R Carmel B, editors. Reptile Medicine and Surgery in Clinical Practice, 1a Edn. New York, NY: Wiley (2017). p. 425–39.

[72] Platt TR. Redescription of *Capillaria serpentina* Harwood, 1932,(Nematoda: Trichuroidea) from freshwater turtles in Virginia. Can J Zool. (1983) 61:2185–9. doi: 10.1139/z83-288

[73] Bay MD Bratt C. Enteric helminth infections in the red-eared slider turtle (*Trachemys scripta elegans*) from southern Oklahoma (2024) 104:105–13.

[74] Easley MS. A Survey of the Parasites of Freshwater Turtles in the Concho Valley. San Angelo, TX: Angelo State University (2022).

[75] Cardells J Garijo MM Marín C Vera S. Helminths from the red-eared slider *Trachemys scripta elegans* (Chelonia: Emydidae) in marshes from the eastern Iberian Peninsula: first report of *Telorchis attenuata* (Digenea: Telorchiidae). Basic Appl Herpetol. (2014) 28:153–9. doi: 10.11160/bah.38

